# Risk of confounding variables in multivariate analysis

**DOI:** 10.1186/s12967-022-03348-0

**Published:** 2022-04-09

**Authors:** Bruno Gagnon, Andrew M. R. Hanna

**Affiliations:** 1 Department of Family and Emergency Medicine, Faculty of Medicine, Université Laval, Quebec City, QC Canada; 2 Cancer Research Centre, Université Laval, Quebec City, QC Canada; 3 Réseau Québécois de recherche en soins palliatifs et de fin de vie du FRQS (RQSPAL), Quebec City, QC Canada; 4 Équipe de recherche Michel-Sarrazin en oncologie psychosociale et soins palliatifs, Université Laval, Quebec City, QC Canada; 5 Division of Palliative Care, Bruyère Research Institute, Ottawa, ON Canada

## Dear Editor,

We too read with interest the article “The Role of Opioids in Cancer Response to Immunotherapy” by Botticelli et al., which suggests that opioids impair the immune response to cancer in patients receiving immunotherapy [1].

First, we are in complete agreement with the concerns raised by Maltoni et Rossi [2] in the first letter you published in response to this article.

Second, while it has been some months since the original article was published, we would like to bring to your attention a new point to consider:

In the Results section, the authors state: “*…opioids use was significantly higher in patients affected by NSCLC [Non-Small Cell Lung Cancer] (p* < *0.0001), in patients with a worse ECOG PS [Eastern Cooperative Oncology Group Performance Status] (p* < *0.0001), in second-line setting subgroup (p* = *0.009), in patients taking corticosteroids (p* < *0.0001), and in patients with a high tumor burden (p* = *0.006)*” [1].

Of those five listed variables, the only one that was not included in the multivariate analysis (MA) was NSCLC. Given that the opioid and non-opioid cohorts consisted of 62% and 22% NSCLC patients, respectively [1], which is an approximately threefold (2.82) difference in proportion, we think that this would have been an important variable to include in the MA. Furthermore, melanoma was overrepresented in the non-opioid group by more than three-fold (3.59; 17% vs. 61% in opioid and non-opioid cohorts, respectively) [1].

The 5-year survival rate of NSCLC, when treated with nivolumab (one of the four immunotherapy drugs included in the study) is 15.6%, compared to 34.2% for melanoma [3]. Tracheal, bronchus, and lung cancers (such as NSCLC) have an age-standardized global mortality rate of 23.74 per 100,000, whereas malignant skin melanoma has a considerably lower rate of 0.78 per 100,000 [4]. Considering these facts, we think that the type of cancer could be a confounding variable that should have been considered in the MA.

If the authors are willing to reanalyze their results, we would be curious to know if and how their conclusions may change if the MA includes the type of cancer. Had the type of cancer been included in the published MA, we suspect that the observed effect of opioids on progression-free survival and overall survival would disappear.

## Data Availability

Not applicable.
